# The Association between Threatened Abortion and the Risk of Autism Spectrum Disorders among Children: A Meta-Analysis

**DOI:** 10.1155/2023/5249585

**Published:** 2023-01-18

**Authors:** Mahshad Ahmadvand, Fatemeh Eghbalian, Shahla Nasrolahi, Ensiyeh Jenabi

**Affiliations:** ^1^School of Medicine, Hamadan University of Medical Sciences, Hamadan, Iran; ^2^Departments of Pediatric, Hearing Disorders Research Center, Hamadan University of Medical Sciences, Hamadan, Iran; ^3^Clinical Research Development Unit of Fatemieh Hospital, Department of Gynecology, School of Medicine, Hamadan University of Medical Sciences, Hamadan, Iran; ^4^Autism Spectrum Disorders Research Center, Hamadan University of Medical Sciences, Hamadan, Iran

## Abstract

**Background:**

The current study is aimed at updating the observational studies on the relationship between threatened abortion and the risk of ASD.

**Methods:**

The search keywords were covered in three electronic databases PubMed, Web of Science, and Scopus up to April 2022. The modified Newcastle–Ottawa scale (NOS) was applied to detect the quality of epidemiological studies. We used the chi-square test and the *I*^2^ statistic to show the heterogeneity among articles. *I*^2^ more than 50% was considered high heterogeneity. Egger's and Begg's line regression tests were used for evaluating the publication bias. The random-effects model was applied for the analysis of the findings. The Stata 13.0 software package was applied for analysis and indicated *p* value less than 0.05 as a significant level.

**Results:**

The pooled analysis reported significant differences between threatened abortion and the risk of ASD in adjusted studies (OR = 1.93; 95% CI: 1.12, 2.73; *I*^2^ = 59.5.0%) and in crude studies (OR = 2.17; 95% CI: 1.46, 2.88; *I*^2^ = 39.5%). The evidence of publication bias was not found.

**Conclusions:**

The findings suggest that threatened abortion is a risk factor for ASD. As a result, screening tools to detect are necessary in mothers facing a threatened abortion.

## 1. Introduction

Autism spectrum disorders (ASD) are an evolutionary neurological disease severely limiting social activity. This condition is becoming more common around the world [1]. These people struggle with verbal and nonverbal communication, adaptation skills, and social interactions. The disease is usually diagnosed in children and causes problems with sensory processing and cognitive functioning in learning [2]. The leading cause of the disease is unknown. Although environmental factors play a role in the disease, there is strong evidence that genetic factors also play a role in ASD [3]. Several studies in this area have been conducted to determine the effects of factors such as maternal obesity, neonatal icterus, gender, maternal age, complications, bleeding during pregnancy, congenital heart disease (CHD), low birth weight (less than 2500 grams), drug use during pregnancy, the presence the umbilical cord around the fetus's neck, preterm labor, intrauterine growth retardation (IUGR), no breastfeeding, and small for gestational age (SGA) [4–8].

The correlation between behavioral and psychiatric assessments and diagnosis and genetic assessments are discussed, along with psychiatric treatment and drug approaches for selecting a medication for treating challenging behaviors or common comorbidities in ASD. Treatment varies depending on the patient's diagnosis, severity, cause, and comorbidities [9].

Preliminary studies recommend ASD screening in children aged 18 to 30 months with the nonsuspected disorder.

On the other hand, early behavioral intervention based on functional behavior analysis appears to improve cognitive, language, and adaptive skills [9]. This disorder is diagnosed based on unusual behavioral evidence, such as persistent defects in social communication and limited and repetitive behavioral patterns [10].

Some studies have found a link between the threat of abortion and the risk of ASD. Threatened abortion has been linked to an increased risk of ASD [11]. However, other studies have found no significant link between threatened abortion and the risk of autism spectrum disorders [12].

Wang et al. conducted a meta-analysis study in 2017. They discovered that threatened abortion is a risk factor for autism [13]. However, they searched until October 2016 and included four studies in the meta-analysis. As a result, the current study is aimed at updating the observational studies on the relationship between threatened abortion and the risk of ASD.

## 2. Materials and Methods

Our meta-analysis was performed based on the PRISMA statement.

### 2.1. Inclusion and Exclusion Criteria

The epidemiological studies were included in the present meta-analysis. Examining the association between threatened abortion and the risk of ASD among children was conducted based on the PICO model:

- Population: pregnant women
- Intervention: threatened abortion
- Comparison: without threatened abortion
- Outcome: risk of ASD among children

The exposure variable and outcome of interest were threatened abortion and ASD, respectively. No language, age, race, publication date, and nationality restrictions were applied. The exclusion studies were meta-analyses and review articles, case reports, experimental articles, and letters to Editor.

### 2.2. Information Sources and Search

The search keywords were covered in three electronic databases PubMed, Web of Science, and Scopus up to April 25, 2022 (threatened abortion, threatened abortions, abortion threat, threatened miscarriage, threatened miscarriages, and ASD (autism spectrum disorders or autism) (Supplementary File (available here))). Also, the references were manually checked for reviewing further studies.

### 2.3. Study Selection

We merged the search findings with the Endnote software reference manager, and duplicate studies were deleted. Also, studies were independently extracted by the two authors (M.A and E.J), and any disagreement between the two authors was solved by the supervisor (F.E). Then, full articles that met the inclusion criteria were synthesized.

### 2.4. Data Extraction

Data extracted from full articles were inserted in the sheet in the Stata software. The data titles were first author, year of article publication, location, study's design, population, controlling for confounding variables, child age, and ASD diagnostic criteria.

### 2.5. Methodological Quality

The modified Newcastle–Ottawa scale (NOS) was applied to detect the quality of epidemiological studies [14]. The NOS consists of 3 items showing participants selection, comparability of ASD and non-ASD children, and outcome assessment. The total quality score is 9 points. Studies with ≥7 scores were considered to be of high quality.

### 2.6. Heterogeneity and Reporting Biases

We used the chi-square test [15] and the *I*^2^ statistic [16] to show the heterogeneity among articles. *I*^2^ of more than 50% was considered high heterogeneity [16]. Egger's and Begg's [17] line regression tests were used for evaluating the publication bias.

### 2.7. Summary Measures

All findings were considered dichotomous variables (threatened abortion and ASD children). Therefore, they were expressed as the odds ratio (OR) with 95% confidence intervals (95% CIs). The random-effects model was applied for the analysis of the findings [18]. The Stata 13.0 software package was applied for analysis and indicated *p* value less than 0.05 as a significant level.

## 3. Results

**Figure 1:**
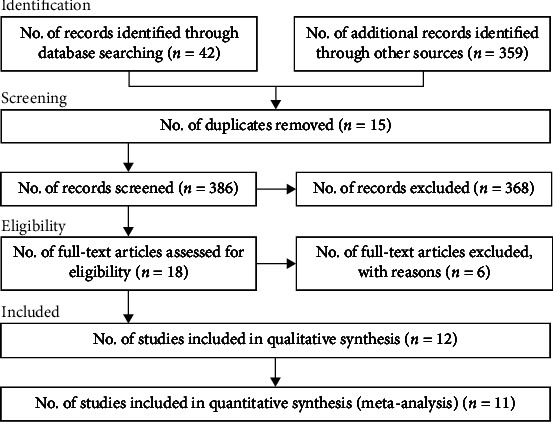
Flowchart of the process selection of the studies.

In total, 401 publications were included until April 25, 2022. Due to duplicate records, 15 were removed. Of these, 368 studies were eliminated after the screening stage (title and abstract reviewing). Eighteen records were included for the last screening (reading the full papers), four of which were deemed ineligible. Finally, 12 studies were included in the present meta-analysis. We identified three cohort studies [5, 19, 20], seven case-control studies [21–27], and two cross-sectional studies [12, 28] (Figure 1). The total population studied was 394,675. All analyses were published in English (Table 1).

Among the included studies, one outlier article was not included in the current meta-analysis because it reported threatened abortion as a protective factor for ASD (OR = 0.35; 95% CI: 0.12, 0.98) [29].

### 3.1. Effects of Exposure

**Figure 2:**
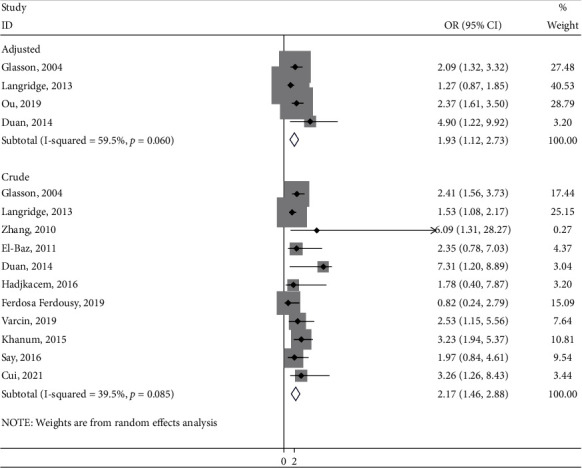
The meta-analysis of the threatened abortion and the risk of ASD.

**Figure 3:**
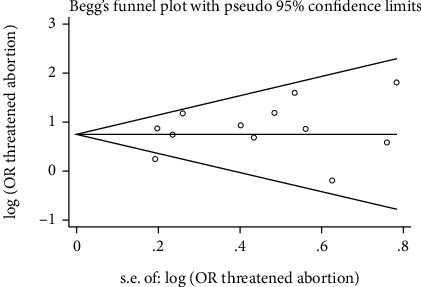
The publication bias of the threatened abortion and the risk of ASD.

Figures 2 and 3 examined the association between threatened abortion and the risk of ASD. The pooled analysis reported significant differences between threatened abortion and the risk of ASD in adjusted studies (OR = 1.93; 95% CI: 1.12, 2.73; *I*^2^ = 59.5.0%) and in crude studies (OR = 2.17; 95% CI: 1.46, 2.88; *I*^2^ = 39.5%) (Figure 2).

### 3.2. Publication Bias

Begg's and Egger's methods were used to detect publication bias. *p* values for Begg's and Egger's regression were 0.891 and 0.323, respectively. The evidence of publication bias was not found (Figure 3).

### 3.3. Quality of the Studies

According to the NOS, six studies were of high quality, while eight were of low quality (Table 1).

## 4. Discussion

The current meta-analysis was based on observational studies examining the association between threatened abortion and the risk of ASD. According to our findings, threatened abortion is a risk factor for ASD.

ASD is one of the reasons for mental retardation in children worldwide. Investigating potential factors may be effective in preventive measures. This association between threatened abortion and the risk of ASD is described by the issue that threatened abortion is related to many developmental disorders in children. It is most likely to be caused by factors such as genetic defects in the fetus, maternal reproductive environment, maternal exposure to toxic, harmful substances, and physical and mental trauma [25]. However, the link between threatened abortion and ASD may be complicated, and the two phenomena may share several common risk factors.

Fetal hypoxia may be at the root of a possible link between threatened abortion due to bleeding and ASD. It has also been reported that hypoxia following a threatened abortion increases dopaminergic activity. Therefore, there is evidence of dopamine overactivation in ASD children [30]. However, potential genetic and environmental factors are possible causes of threatened abortion.

Wang et al. conducted a systematic review of the relationship between prenatal, perinatal, and postnatal factors with autism in 2017 [13]. Threatened abortion was found to be a risk factor for ASD (RR = 2.28; CI: 1.63, 3.19). They searched until October 2016 and included four studies with a sample size of 2249 participants. Therefore, this issue may increase random bias.

One outlier study [29] was not included in the current meta-analysis because it reported threatened abortion as a protective factor for ASD. It was an unmatched case-control study that compared 56 children with ASD to 85 controls. Threatened abortion was found to be a risk factor for ASD (OR = 0.35; 95% CI: 0.12, 0.98). In addition, the quality of this study was poor. The small number of ASD children and the unmatched case-control study raise the possibility of random error, which could explain the difference between the two groups.

## 5. Limitations

Some limitations cannot be controlled by the authors. The majority of the studies were of low quality and had not been adjusted for confounding variables. Also, we must consider the possibility that the missing data from the gray literature will affect the overall effect estimates.

## 6. Strength Points

According to the current meta-analysis, which included 394,675 participants, threatened abortion is a risk factor for autism spectrum disorders in children. In addition, evidence of publication bias was not found.

## 7. Conclusion

The findings suggest that threatened abortion is a risk factor for ASD. As a result, screening tools to detect are necessary in mothers facing a threatened abortion.

## Figures and Tables

**Table 1:** Characteristics of the included studies in the present meta-analysis.

1^st^ author, publication year	Country	Design	Sample	Diagnose method for ASD	Child age (year) (mean or range)	Estimate	Adjustment	Quality
Glasson [5]	Australia	Cohort	1778	ICD-9	<3	OR	Crude/adjust	High
Langridge [19]	Australia	Cohort	383153	DSM-IV-TR	Not reported	OR	Crude/adjust	High
Zhang [26]	China	Case-control	190	ICD-10/CARS	3-21	OR	Crude	Low
El-Baz [22]	Egypt	Case-control	200	DSM-IV	2-13	OR	Crude	Low
Ou [25]	China	Case-control	2306	DSM-IV-TR	Not reported	OR	Adjust	High
Duan [21]	China	Case-control	572	DSM-IV/CARS	Healthy:4.14 Autism:4.26	OR	Crude/adjust	High
Hadjkacem [12]	Tunisia	Cross-sectional	101	CARS	3-12	OR	Crude	Low
Fatema Ferdousy [23]	Bangladesh	Case-control	200	ADOS	2-6	OR	Crude	Low
Varcin [20]	Australia	Cohort	1238	Not reported	19-20	OR	Crude	High
Khanum [24]	Bangladesh	Case-control	297	Not reported	5-7	OR	Crude	Low
Say [26]	Turkey	Case-control	180	DSM-IV	3-18	OR	Crude	Low
Cui [28]	China	Cross-sectional	318	DSM-IV	2-6	OR	Crude	Low

ASD: autism spectrum disorders; OR: odds ratio; ICD: International Classification of Diseases; DSM: Diagnostic and Statistical Manual of Mental Disorders; CARS: Childhood Autism Rating Scale; ADOS: Autism Diagnostic Observation Schedule.

## Data Availability

Access to data is possible with permission from the responsible author.

## Abbreviations

NOS: Newcastle–Ottawa scale
ASD: Autism spectrum disorders
PRISMA: Preferred Reporting Items for Systematic Reviews.

## Supplementary Materials

Supplementary Materials Strategy search.
