# Supplementary material for: The Association between Threatened Abortion and the Risk of Autism Spectrum Disorders among Children: A Meta-Analysis
Source: Biomed Res Int. 2023 Jan 18;2023:5249585. doi: 10.1155/2023/5249585 (PMC9876674; doi:10.1155/2023/5249585)

**Supplementary : Strategy search**

**PubMed**

**Search: ((((((threatened abortion[Title/Abstract]) OR (threatened abortions[Title/Abstract])) OR (abortion threat[Title/Abstract])) OR (threatened miscarriage[Title/Abstract])) OR (threatened miscarriages[Title/Abstract])) OR (abortion threatened[MeSH Major Topic])) AND (((autism spectrum disorder[MeSH Major Topic]) OR (((ASD[Title/Abstract]) OR (autism spectrum disorders[Title/Abstract])) OR (autism[Title/Abstract]))) OR (((ASD[Title/Abstract]) OR (autism spectrum disorders[Title/Abstract])) OR (autism[Title/Abstract])))**

**SCOPUS**

( ( TITLE-ABS-KEY ( threatened  AND abortion ) )  OR  ( TITLE-ABS-KEY ( threatened  AND abortions ) )  OR  ( TITLE-ABS-KEY ( abortion  AND threat ) )  OR  ( TITLE-ABS-KEY ( threatened  AND miscarriage ) )  OR  ( TITLE-ABS-KEY ( threatened  AND miscarriages ) ) )  AND  ( ( TITLE-ABS-KEY ( asd ) )  OR  ( TITLE-ABS-KEY ( autism  AND spectrum  AND disorders ) )  OR  ( TITLE-ABS-KEY ( autism ) ) )

Web of Science


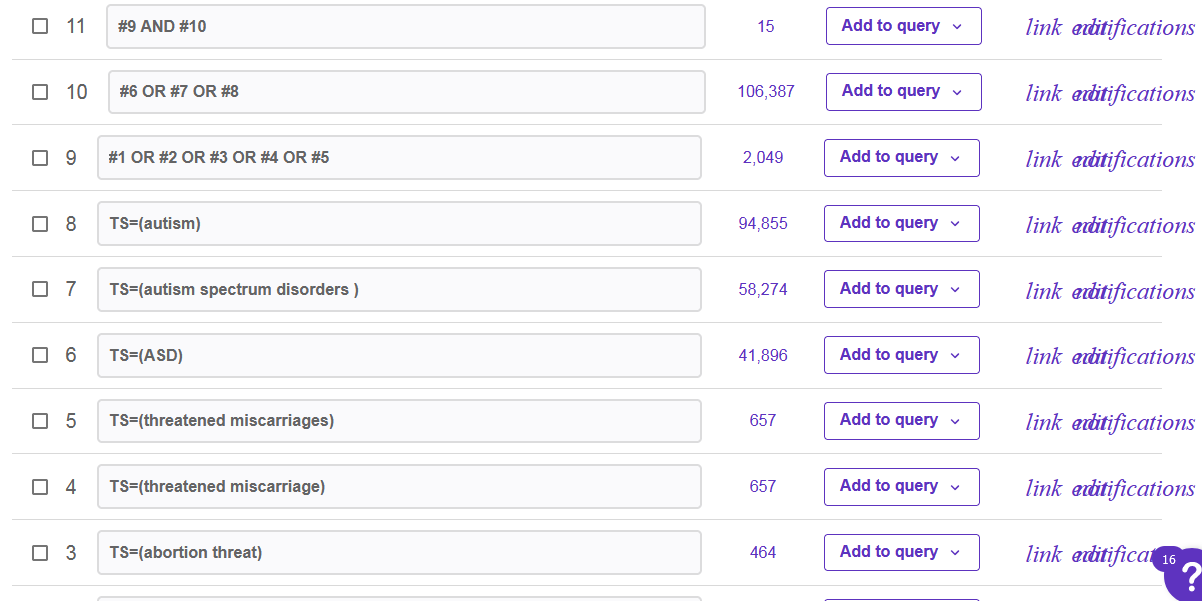

Supplement: Supplementary Materials — Strategy search. [file 5249585.f1.docx]
